# How can end of life care excellence be normalized in hospitals? Lessons from a qualitative framework study

**DOI:** 10.1186/s12904-018-0353-x

**Published:** 2018-08-08

**Authors:** Christy Noble, Laurie Grealish, Andrew Teodorczuk, Brenton Shanahan, Balaji Hiremagular, Jodie Morris, Sarah Yardley

**Affiliations:** 1 Medical Education Unit, Gold Coast Health, Level 2 PED Building, 1 Hospital Boulevard, Southport, QLD 4215 Australia; 2 School of Medicine, Griffith University, Griffith, QLD Australia; 3 School of Pharmacy, University of Queensland, Brisbane, QLD Australia; 4 School of Nursing and Midwifery and Menzies Health Institute Queensland, Griffith University, Griffith, Queensland Australia; 5 Gold Coast Health, Griffith, QLD Australia; 6 Myton Hospices, Coventry, UK; 7 Central and North West London NHS Foundation Trust, London, UK; 8 Marie Curie Research Department, University College London, London, UK

**Keywords:** Palliative care, Hospital, Qualitative research, Normalisation process theory, Guideline

## Abstract

**Background:**

There is a pressing need to improve end-of-life care in acute settings. This requires meeting the learning needs of all acute care healthcare professionals to develop broader clinical expertise and bring about positive change. The UK experience with the Liverpool Care of the Dying Pathway (LCP), also demonstrates a greater focus on implementation processes and daily working practices is necessary.

**Methods:**

This qualitative study, informed by Normalisation Process Theory (NPT), investigates how a tool for end-of-life care was embedded in a large Australian teaching hospital. The study identified contextual barriers and facilitators captured in real time, as the ‘Clinical Guidelines for Dying Patients’ (CgDp) were implemented. A purposive sample of 28 acute ward (allied health 7 [including occupational therapist, pharmacists, physiotherapist, psychologist, speech pathologist], nursing 10, medical 8) and palliative care (medical 2, nursing 1) staff participated. Interviews (*n* = 18) and focus groups (*n* = 2), were audio-recorded and transcribed verbatim. Data were analysed using an a priori framework of NPT constructs; coherence, cognitive participation, collective action and reflexive monitoring.

**Results:**

The CgDp afforded staff support, but the reality of the clinical process was invariably perceived as more complex than the guidelines suggested. The CgDp ‘made sense’ to nursing and medical staff, but, because allied health staff were not ward-based, they were not as engaged (coherence). Implementation was challenged by competing concerns in the acute setting where most patients required a different care approach (cognitive participation). The CgDp is designed to start when a patient is dying, yet staff found it difficult to diagnose dying. Staff were concerned that they lacked ready access to experts (collective action) to support this. Participants believed using CgDp improved patient care, but there was an absence of participation in real time monitoring or quality improvement activity.

**Conclusions:**

We propose a model, which addresses the risks and barriers identified, to guide implementation of end-of-life care tools in acute settings. The model promotes interprofessional and interdisciplinary working and learning strategies to develop capabilities for embedding end of life (EOL) care excellence whilst guided by experienced palliative care teams. Further research is needed to determine if this model can be prospectively applied to positively influence EOL practices.

## Background

Providing high-quality care for dying patients in acute settings is essential to meet changing population needs and societal expectations [1]. Increasingly people live with chronic, potentially life limiting conditions; prognosis is uncertain and inevitably some will spend their final days of life in acute hospital care [2, 3]. In Australia, despite 70% of people wanting to die at home only about 14% do [3, 4]. While healthcare organisations are adopting care pathways to ensure appropriate end of life care, globally, studies report suboptimal care quality in hospitals [5–7].

For many years, the Liverpool Care Pathway (LCP) was considered as a gold standard for care for dying patients, yet it has been withdrawn in the United Kingdom (UK). Significant gaps were identified between the goals of the LCP and its enactment in practice, with criticism focused on failure to have studied the process of implementation, lack of available expertise embedded in acute settings and not addressing the learning needs of non-specialist healthcare professionals [8]. As a result five priorities for improving care for a dying person [9] were identified: a) recognising the possibility of dying; b) sensitive communication with the dying person and loved ones; c) involve dying person and loved ones in treatment and care decision making; d) explore and respect the need of the dying person’s loved ones and e) agree and deliver an individualised care plan with compassion [9]. Contrasting with the UK experience, Italian studies suggest that the quality of end of life (EOL) care can be improved using the LCP when implemented with a structured program and clear goals [5–11]. These findings align with emerging evidence suggesting that effective LCP implementation can be aided by a comprehensive understanding of the local context including appreciating and valuing differing professional perspectives and work structures [10, 11].

Achieving high-quality end-of-life care in acute settings is challenging. [6] Acute settings are orientated towards interventional treatment of reversible conditions. Institutions are designed to ensure rapid provision of immediate and urgent care with finite specialist palliative care resources, staff turnover is often high and staff not necessarily experienced in recognising dying, or determining likelihood of reversible causes versus irreversible deterioration [4] despite multiple attempts to educate and improve [5]. Enabling excellence in EOL care in acute care settings is a significant concern, with both national [12] and international [9] strategies being developed to support these provisions of EOL care excellence.

This study aimed to investigate if and how EOL care excellence can be embedded or normalised in acute healthcare settings. Our objectives were to: 1) generate a rich description of individual and contextual barriers and enablers surrounding implementation of the Clinical Guidelines for Dying Patients (CgDp), in an acute setting; 2) identify learning strategies to mitigate barriers and strengthen enablers; 3) integrate our data analysis, using normalization process theory (NPT) (see below), to generate a conceptual model to inform further implementation research.

## Methods

### Theoretical framework

We adopted a social constructionist research approach [13] to understand the meanings participants generated through interactions when enacting practices related to the CgDp. Normalisation Process Theory (NPT) [14, 15] was used as our theoretical lens as it offers an explanation of processes for implementing, embedding and integrating practices in everyday work [14]. Individual and contextual factors promoting or inhibiting normalisation of practices include coherence, cognitive participation, collective action and reflexive monitoring (see Table 1 for further explanation of each construct). This approach enabled us to develop in-depth descriptions and interpretative explanations for how learning strategies can facilitate the CgDp becoming integrated in practice.

**Table 1:** Normalisation process theory constructs - generative mechanisms (From May et al., 2009 [37])

NPT construct	Explanation
Coherence	Work that defines and organises the objects of a practice
Cognitive participation	Work that defines and organises the enrolment of participants in a practice
Collective action	Work that defines and organises the enacting of a practice
Reflexive monitoring	Work that defines and organises the knowledge upon which appraisal of a practice is founded

### Study design

Given the need to understand local contextual factors for effective implementation [8, 14], an explanatory qualitative interview study, using the framework analytic approach [16], was conducted that aligned to our theoretical stance. Our design facilitated in-depth exploration into the health care professionals’ perspective of individual and contextual barriers and enablers to implementing the CgDp. NPT as a sensitising tool for embedding practices [17] enabled us to generate a programme theory and model [18] to inform the development of a complex intervention to normalise end of life care excellence in acute care settings. The study was approved by Gold Coast Health Ethics Committee (HREC/14/QGC/185). To ensure the anonymity of the participants and the practices where they work, all identifiers have been removed. Written consent was obtained.

### Setting

This study was conducted in a large acute care tertiary hospital, located in Queensland, Australia with 750 inpatient beds. The study focussed on a 24-bed acute care medical ward, where there were 48 deaths in 2015. In 2009, an EOL care pathway, CgDp, based on LCP was endorsed by Queensland Health for state-wide use within its acute hospitals. [19] (The current version of the CgDp can be found here: https://www.health.qld.gov.au/clinical-practice/guidelines-procedures/patient-safety/end-of-life/guidelines along with further information about the documents and implementation strategy of Queensland Health plus their contact details. The document has undergone minor revisions since the study was conducted). In 2013, acute care providers were charged with providing local education and audits to support implementation of the pathway. However, acute ward hospital staff reported that, from their perspective, the CgDp had ‘appeared’ on the wards with limited formal training provision. Efforts to address this need provided the opportunity for this study to capture ‘real world’ acute care clinician challenges as they integrated the CgDp into acute practice settings. Moreover, it offered an opportunity to systematically identify strategies to normalise EOL care excellence in a typical acute care setting.

### Participants

Purposive sampling [20] was used to ensure recruitment of a range of perspectives within professional stakeholders, including the nursing, medical and allied health professionals and the palliative care team. Staff were identified through discussion with nursing, medical and allied health staff leaders and participants were recruited in the following ways. For allied health staff, the Directors of each professional group were emailed and asked for the details of the clinician working in the study ward. Seven clinicians were identified and contacted by the study team via email and all agreed to participate in the study. For the medical staff, the researchers presented an overview of the study at their team meeting (*n* = 17) and 8 agreed to participate. For nursing staff, because of shift work, the research team attended two nursing handovers to present an overview of the study and placed posters in the tea room. It is not known how many nursing staff viewed the poster or missed the handover meeting. For palliative care staff consulting to the study ward, we directly contacted, via email and follow up phone call, the consultant, advanced trainee and clinical nurse consultant and all agreed to participate.

### Data generation

Qualitative, semi-structured interviews, individual or group, depending on participant availability, were conducted. The interviews, conducted from June to August 2015, explored perceptions, views and experiences of caring for dying patients and how the CgDp was used. Interviews were conducted by the authors who did not work in the study (ward) setting. The interviews occurred face to face in private rooms and were planned so that there was no conflict of interest or perceived hierarchy between interviewer and interviewee. All interviews were audio recorded, transcribed verbatim and rechecked for accuracy. Data collection continued until the research team agreed that theoretical saturation was reached; that is, additional data was not anticipated to produce new perspectives or insights [20].

### Data analysis

Data were analysed using a five phase framework method: 1) familiarisation, 2) identifying a thematic framework, 3) indexing, 4) charting and mapping and 5) interpretation [16]. Research team members who conducted the interviews (CN, LG, BS, JM), and led on the analysis (SY, AT) familiarised themselves with raw data and discussed their impressions of the dataset. The NPT constructs provided the a priori thematic framework (See Table 1). Next, the transcripts were divided amongst members of the research team (CN, LG, JM, BS and BH), and each transcript was independently coded or indexed, using the framework, by two researchers who then discussed the coding in pairs and to negotiate agreement. Once the transcripts were coded and the data were charted and mapped in Excel®, themes were agreed upon. This coding and theming was independently reviewed and confirmed by AT and SY. The key themes were presented to the participants, who verified these themes, as part of two usual ward meetings. Patterns and associations between themes were interpreted and used to develop a mid-range theory model to inform further implementation strategies.

## Results

**Fig. 1:**
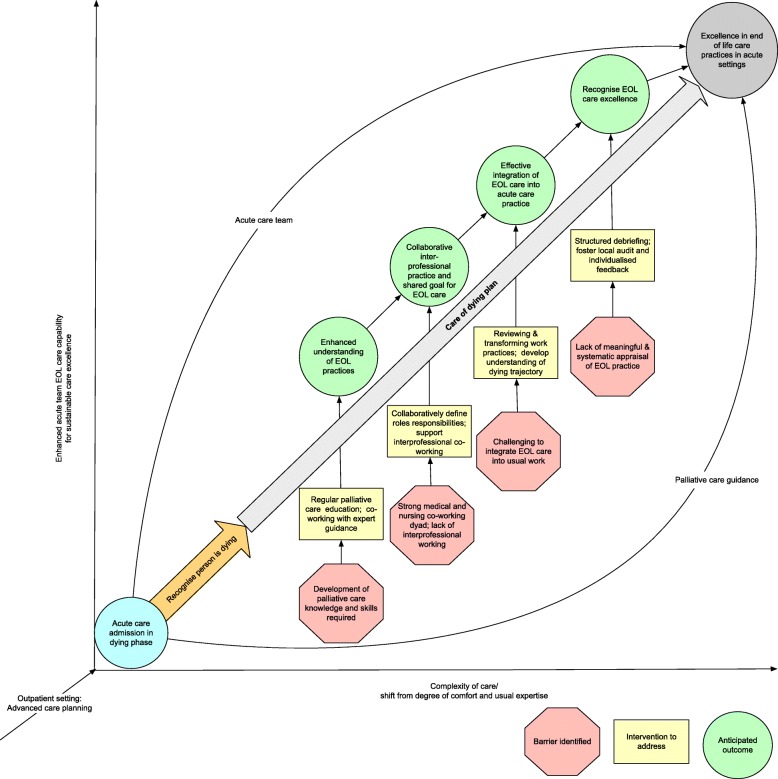
Proposed implementation model for augmenting EOL care excellence in acute care settings. The x-axis represents increasing EOL care complexity and shift from degrees of comfort and usual expertise. The y-axis represents the development of the acute care team’s capability towards sustainable EOL care excellence. The central diagonal arrow represents CgDp which supports EOL care practices. The engagement of the acute care team and palliative care team are symbolised by the curved arrows and illustrates that as the acute care team develops in EOL care capability their ability to provide EOL care excellence in less complex cases is enhanced without extensive palliative care guidance. While for more complex cases, the guidance of palliative care called upon. The barriers identified, based on the NPT constructs, are presented along with interventions to address these and the anticipated outcomes, which when combined are likely to contribute to excellence in EOL care practices in acute settings

Twenty-eight professionals were interviewed, in 18 individual and two group interviews, including allied health (7) (occupational therapist, pharmacists, physiotherapist, psychologist, speech pathologist), nursing (10), medical (8) and palliative care (medical, 2; nursing, 1). The average time for the interviews was 36 min (range: 18–55 min). The results are presented in two parts: 1) based on the NPT constructs, the key barriers and enablers identified (Table 2) 2) implementation and learning model, including accompanying learning strategies (Table 3). A mid-range programme theory, based on these findings, to inform strategies for augmenting EOL care excellence in acute care settings is developed from analysis of the findings (See Fig. 1).

**Table 2:** Key barriers and enablers

Normalisation Process Theory Construct	Key enablers	Key barriers
Coherence (what is the work)	• CgDp signals a shift to a different type of care • CgDp valued by staff as it supports systematic approach to end of life care • CgDp legitimises caring for the dying in acute setting	• The need for CgDp suggestive of a failure in acute care provision • Lack of education and training in principles of palliative care and care of the dying • Professionals conceptualise CgDp as ‘everything’ or ‘nothing’ because challenged by uncertainty posed when variances or individualised care was required
Cognitive participation (who does the work)	• CgDp empowers nursing staff to discuss EOL care with medical staff • Guidance available from palliative care team • Clear lines of responsibilities e.g. medical team lead decision making • Medical profession willing to lead implementation of CgDp intervention • Recognition that effective patient and family communication required	• Lack of genuine multidisciplinary team working • CgDp being enacted without an interprofessional approach • Lack of understanding of roles related to CgDp • Allocation of roles and responsibilities tend to mirror acute practice roles (not recognising that a different approach is required e.g. MDT) • Usual expert guidance structures challenged because EOL care not usual part of practice
Collective action (how does the work get done)	• Familiar with CgDp documentation • Effective collaboration between nursing and medical staff • Established relationships with patients • Nursing staff creating environment conducive to EOL care • Palliative care team provides decision making support e.g. diagnosing dying; symptom management advice • Continuity of care within speciality considered to be important e.g. home-ward • Mentoring and learning occurring through practice	• Challenging to integrate effective EOL care in the context of acute setting (e.g. organisational pressures for discharge) • EOL care provision infrequent activity • Allied health tendency to disengage in and/or excluded from EOL care • Senior medical officers not fully engaged in CgDp intervention e.g. delegate to juniors • Absence of allied health engagement • Documentation considered burdensome and not aligned to technology e.g. electronic medical records • Lack of longitudinal palliative care planning resulting in reactive response to dying patients • Rostering and staffing arrangement hamper allied health and palliative care not able to fully integrate and support
Reflexive monitoring (how is the work understood and changed)	• Desire to integrate/improve EOL decision making processes • Recognition that structured debriefing sessions are required improve quality of CgDp care	• Systematic audit and feedback processes required to inform and improve outcomes • Few opportunities for meaningful clinical supervision to provide emotional support • Staff find it challenging to find ways to meaningfully appraise the effectiveness of CgDp practices and outcomes

**Table 3:** Proposed learning strategies to embed EOL care excellence

Normalisation Process Theory Construct	Proposed learning strategies	Examples
Coherence (what is the work)	• Support development of palliative care knowledge and skills • Facilitate expert guidance for staff in situations of uncertainty e.g. feedback from palliative care on performance	• Regular education programs supporting the development of all acute care staff (including rotational and locum) • Interprofessional team learning, in collaboration with palliative care team where real-life scenarios are explored
Cognitive participation (who does the work)	• Foster an interprofessional approach to EOL decision making and care provision through learning activities • Define duties and responsibilities of health care staff • Promote interprofessional working and learning • Palliative care to provide guidance on interprofessional approaches to EOL care	• Develop and implement interprofessional learning activities to support EOL practices including practice-based or simulation activities
Collective action (how does the work get done)	• Review work structures, rostering and processes to support prioritisation of EOL care • Educate staff on the long trajectory required for effective EOL care • Create learning programs that challenge assumptions about roles and accepted ways of working • Augment opportunities for co-working with palliative care team • Simulated interprofessional learning experiences	• Prioritise dying patients on ward rounds • Integrate EOL practices into outpatient setting e.g. have a checklist; review outpatient list who had multiple admission • Interprofessional case-based discussions – range of contexts e.g. outpatients; acute setting
Reflexive monitoring (how is the work understood and changed)	• Support development of self-regulation on individual practices • Enhance opportunities for audit and feedback • Create opportunities for staff to debrief as a team	• Schedule regular ‘after death’ care reviews for multidisciplinary team with guidance from palliative care team

### Coherence – Making sense of the CgDp

Participants agreed the CgDp’s purpose made sense as a support for effective EOL care, yet they experienced tensions when attempting to integrate EOL practice into their daily work. For example, participants indicated that using the CgDp provided evidence to legitimise EOL care provision in acute settings and increased confidence to take action. This was described in the following extract by a nurse who explained:

… That [the pathway] gives us a bit more reassurance that we can do less [acute care] and focus more on comfort and quality… They just want to cover themselves legally I think. (Nurse-3).

Hence it appeared that the pathway ‘gives permission’ to shift the focus of care from a more traditional biomedical model to a more holistic approach. Despite this permission, based on their understanding of their role in acute care, other people felt that EOL care lacked alignment with this role:

I don’t want to use them [CgDp]…we are an acute ward…our patients are meant to recover… (Nurse-5).

In this extract, the nurse suggests that using the CgDp is somewhat at odds with her role identity which defines success as cure.

The CgDp did not account for the uncertainties and complexities participants experienced when engaging in EOL care. Participants found recognising dying a particularly challenging aspect. Reasons were multifactorial including a lack of experience and/or guidance; not understanding palliative care principles; experiencing diagnostic uncertainty and avoiding EOL care decision making and/or worrying the wrong decision has been made; wanting to exhaust all treatment options before commencing CgDP and finding the process personally uncomfortable. This is captured in the next extract by an allied health professional:

I didn’t pick up the signs of that last patient that I had that was actually passing away. Ahh! They weren’t on a care of the dying pathway, so I still treated them actively, which I felt at the time was appropriate. On reflection…maybe I didn’t add any benefit to it. I don’t think I made it any worse, at least I hope I didn’t. (Allied Health-2).

While the CgDp increased awareness of the need for EOL care in acute settings, this was perceived as a challenge rather than empowerment. The CgDp as an artefact introduced into the setting created disruption in routines and consequently staff had to consider the possibility of dying and decide if this might be the case but they found it challenging to do make such a decision. This is reflected in the next extract when two nurses within a focus group outline the difficulty, experienced by doctors, in deciding when a patient is at the point to need transfer to the CgDp:

Nurse-1: It’s like they’re [doctors] are scared of it.

Nurse-2: …as doctors they’re taught to save lives and then when they’ve got to do this thing [CgDp] where they’re basically withdrawing treatment to let somebody die, they worry that they are doing the wrong thing. I think they need to be told that it’s okay to do this. This is a good thing for them. (Group interview 1-nursing).

Participants described uncertainty with the duality of aiming to ‘reverse the reversible’ while holding the possibility of dying and appropriate CgDp care alongside this. In contrast, participants felt much more comfortable when they could focus solely on one form of practice e.g. active treatment or EOL care:

So to give you an idea, we have had elderly patients who come in with bad pneumonia and are possibly dying, not responding to treatment, and the question is do you continue with full measures, such as antibiotics, fluids et cetera or do you just focus on comfort care? (Medical officer-10).

Participants agreed that the CgDp provided guidance on priorities when caring for dying patients. This was reassuring to staff, especially to novice practitioners:

So, it’s more like a guideline. So, it’s more of a checklist for everyone making sure that we don’t miss things you know. So, it’s a cross multidisciplinary thing. (Medical officer-5).

Overall, participants understanding of the purpose of the CgDp in acute care, was that it was to systematise EOL care. However, the key challenges to EOL care excellence were uncertainty when diagnosing dying, appropriately individualising patient care, and difficulties experienced integrating EOL care practices with acute care practices. These findings are presented in Table 2.

### Cognitive participation – Getting involved in CgDp

Contrary to the CgDp goal, of providing uniformly good, albeit individualised, care to dying patients, using the guidelines did not disrupt parallel working between professions to achieve fully the integrated multidisciplinary and interprofessional working required. Working in parallel, profession-specific lines meant that some professional groups lacked certainty regarding the legitimacy of their engagement. For example, the decision to commence the CgDp was consultant (senior physician) led, with prompting from nursing staff but little to no engagement from allied health practitioners (AHP). The following extract outlines how AHPs perceived they were potentially excluded from these processes:

I don’t know if I should be doing it [CgDp]. I know there’s an allied health section, but I was never really told to or asked to write in the forms, so I never have. (Allied Health-7).

However, using the CgDp promoted certain staff to reflect on and change their existing ways of working. Some nursing staff felt empowered to prompt the medical team to make decisions about EOL care and played a role in reassuring medical staff about their EOL care decision. However, they were often frustrated when waiting for the CgDp paperwork to be completed by medical staff as illustrated in the following extract:

Interviewee 3: The doctors not filling stuff out.

Interviewee 4: Sometimes it could take 24 h for a doctor to do it.

(Group interview 1-nursing)

Nursing staff were concerned this delayed multiprofessional provision of holistic EOL care.

Moreover, some senior nursing staff noted that because patients infrequently died on the ward, they needed to reorient themselves to the practices associated with the CgDp:

Yes I’m probably a bit shaky on it for myself because I haven’t actually used it for quite a long time. (Nurse-3).

This meant usual patterns of co-working e.g. seeking guidance from more senior staff differed for practices associated with the CgDp from usual acute care practices.

The perceptions of medical staff, recognising their need for decision-making reassurance sometimes sought guidance from the palliative care team, usually via telephone consults. Despite this, aspects of EOL care, including completing the CgDp paperwork, were delegated by senior medical staff to their juniors. Thus, some consultants (senior physician) avoided direct engagement with practical complexities required for effective enactment of the CgDp, and did not always appear to have insight into these, as described by a consultant (senior physician):

I know about it [CgDp] but I don’t know …the details… probably because I’m confident that I know when a patient is near reaching their end stage I don’t need to look for any guidelines to help me. (Medical officer-6).

Additional care strategies were sometimes triggered and adopted instead of the CgDp when complexities became explicit, such as a so-called, ‘trial of life’ where full treatments were applied for 24 to 48 h to determine if patients might improve. This is described by a consultant below:

If there’s anything that you felt like, oh look, you know, he’s not - we’ll give him a trial of 24 to 48 h, for example, and there’s still no progression and still no improvement and having a chat with the family and things you know say, look if that’s not improving I think it’s for comfort measures and then that’s the time that we’ll be looking at the realm of like the Care of the Dying Pathway. (Medical Officer-5).

This suggests an ‘either or’ conceptualisation of active disease treatment versus palliation remained suggesting that further opportunities for learning about palliative care principles are necessary for effective enactment. In particular, education should include individualised decision-making and the appropriateness of multifaceted approaches when situations are uncertain i.e. using CgDp alongside interventional treatments.

Lastly, the strength of medical and nursing dyad created a barrier to AHP engagement and decision making when the CgDp commenced. All AH participants indicated that they were willing and able to contribute to EOL care. Moreover, they noted that the CgDp specifies the AHP contributions (as above), however, they expressed concern that medical and nursing teams did not understand how they might contribute to EOL care:

Just someone say, we [nursing and medical staff] want you to do it, because at the moment no one is saying that we need to do it or want us to do it, so we’re not going to do something that is going to probably take up more time if we’ve never been asked to do it…if we sat down and it was said that – a ward says we want everyone to be involved in this, in using this pathway [CgDp], so we work together, then for sure, I wouldn’t mind. I’d be more than happy to be involved. (Allied Health-1).

### Collective action – Implementing CgDp

Effectively enacting the CgDp in a busy acute setting was challenging and barriers to effective EOL care provision included: 1) an over reactive response to EOL care rather than proactive or long-term i.e. advance care planning and 2) privileging of acute care practices and work. Combined, these two factors created a sense of being overwhelmed faced with the work associated with attending to dying patients’ needs as exemplified in the following extract by a medical officer:

But our days are busy, so I figure there’d almost be a bit of a delay sometimes, because for instance we did one ward round this morning, we finished at 11:00. Then the senior doctors had to go to the radiology meeting until about 12:00. Then comes the next consultant for the next ward round. So, at what point do you have that time to throw that paperwork [CgDp] together as well. It can be quite difficult. (Medical officer 9).

The reactive response, as opposed to proactive (i.e. advance care planning), and lack of prioritisation related to perceptions that CgDp required a complex cascade of actions, many of which fell outside of participants’ comfort zone e.g. family meetings, completion of paperwork. This response could also be attributed to complex and busy work practices, staff resourcing and lack of palliative care training. For example, the medical team needing to attend to patients on other wards; allied health work scheduling meant they were not ward-based and therefore not fully integrated into ward practices. Thus, missed opportunities, because some allied health staff were reactively rather than collaboratively invited to participate, existed for appropriate skill mix and building relationship with patients and family members:

…it depends on how much rapport you’ve built with the family I think. I’ve been thrown in a few where there’s such - on the ward with the nursing staff and the doctors it’s, he or she is going to pass away, quick, get in there, and the moment you get in there the family is like, who are you. (Allied health-5).

Several system-level issues created barriers to EOL care including: 1) organisational pressure for patient discharges; 2) lack of opportunity for meaningful palliative care team guidance and 3) paper-based CgDp guidelines in the context of electronic medical records. Each of these barriers are explored below. Firstly, the organisational pressures being experienced are described here:

There is always someone from the top calling you to discharge the patients…and there is not time to go through [the dying conversation] when they are not dying…for example, with chronic obstructive airways disease exacerbation, come in, get steroids and antibiotics, go home… there is no time to talk about dying then. When they come back with acute illness and are dying… all of a sudden someone will ask, where is their resuscitation plan…there’s no plan so you press the panic button …it’s a big mess. (Medical officer-2).

Secondly, participants appreciated palliative care guidance, however, obtaining this guidance was a challenging endeavour due to both teams’ busy workloads. Thus, often resorting to telephone conversations rather than face to face collaborations. Palliative care staff were also uncertain if their advice was always sought when needed:

I think some consultants must have their own views on how the care should be given for a dying patient and I guess they must think that they know. You can’t be an expert in every area and I think it’s important enough not to think that you could do everything and then blind yourself to the mistakes that you’re making. (Palliative care medical officer-4).

Thus, in terms of collective action, some palliative care team members perceived that their contributions were not being acted on.

Thirdly, a further crucial contextual factor was that the hospital uses electronic medical records and the CgDp was paper based. This meant that staff who defaulted to the electronic medical records were not always aware of the decision to commence the CgDp or its progress. Moreover, the CgDp was a large document, which combined with infrequent use became perceived as challenging to complete. These views contrasted with palliative care staff who indicated that despite its size, with practice, the document was relatively easy to engage with.

### Reflexive monitoring –embedding and improving CgDp

Embedding EOL care in acute settings requires staff reflection on the implementation of the CgDp and where necessary amending of practices to ensure best outcomes. Despite having the CgDp as a practice guide, the participants indicated further improvements in EOL care were required. Barriers to effective staff reflection and improvement were identified and these included firstly, a lack of structured and meaningful team debriefing to improve EOL care. Secondly, a lack of knowledge and skills to meaningfully appraise EOL care and its outcomes. Finally, a lack of systematic audit and feedback processes to inform and improve EOL care outcomes.

While some staff indicated that they informally debrief after a patient death with peers, there was an absence of interprofessional and structured debriefing. Attempts at changing EOL practices seemed to be largely speculative and participants were not able to describe ways that they were changing or had changed their EOL practices as a team. This meant that important ways to improve EOL care were left unresolved:

I guess the one thing we don’t do more of, or I don’t do more of, is to actually talk about dying much earlier on. So, we don’t have a lot of that. So that discussion sort of comes about in context of Care of the Dying Pathway, which may not be ideal. I’m not sure if it is the right thing to be discussing these things early on. I mean, there’s some evidence to say that that’s what patients want to know and we probably should do that. But we haven’t been, mainly because we don’t have a structure for that; who does it and when, do you do it in hospital or clinic? (Medical officer-10).

In these ways, this medical officer recognises the reactive responsive EOL care whilst acknowledging that integrative of EOL care planning had not been addressed.

A key factor hampering staff ability to meaningfully reflect on and improve EOL care was a lack of expertise and experience in palliative care. This meant it was challenging to recognise the features and enact EOL care excellence as evidenced by the next extract:

But we don’t know what to compare it [EOL care] to. I don’t know if anybody else has worked in a hospice or palliative care where would it be just - could it be not as busy but it could be a lot of noise and trolleys and things trundling up and down and bells going off you know. (Nurse-5).

Since comprehensive audit and feedback processes to inform and improve outcomes related to effective implementation of CgDp were absent, the effectiveness of EOL care was not apparent to participants. As such they were not receiving individualised feedback on the quality of their care nor were they aware of the quality standards to be achieved. Many participants noted that quality EOL care is not being wholly addressed through audit processes:

I mean, we do have deaths in hospital but the kind of deaths we have on our ward are ones that are fairly catastrophic, so where people have had MET calls and had cardiac arrests and gone to ICU [intensive care unit] and then died in ICU. They are few and far between and we review them in our morbidity and mortality meetings and almost always there is nothing that could have prevented that outcome that we can pick up and it’s just the natural course of the illness. Then occasionally we have the patients who have come in, not improved with treatment and have just deteriorated and in those situations they are often patients that we know have severe illness and for them that would seem very appropriate. In fact, you could even argue not starting Care of the Dying Pathway was probably inappropriate and we’re just continuing their agony and misery. (Medical officer-10).

### Development of a theoretical model for education and implementation

Figure 1, presents a proposed implementation model for augmenting EOL care excellence in acute care settings. The model, presents the key barriers to effectively embedding EOL care in acute settings. In response to these barriers, learning and implementations strategies have been identified which are likely to contribute to EOL care excellence and to sustain these practices [21, 22]. Recognising the complexity of EOL practice, guidance and support from palliative care is recommended [23]. However, as the acute teams’ EOL capability is augmented and the key learning outcomes are achieved, guidance is likely to be limited to the most complex cases. This model provides lessons for implementation for acute care settings who are implementing and/or revising their EOL care practices. Additionally, this model presents an EOL learning curriculum for HCP working in acute setting using a range of practice-based pedagogic strategies.

## Discussion

This study sought to generate a conceptual model of the realities of embedding an end-of-life-care tool into an Australian acute hospital. Our research team were cognisant of the challenges this presented from international experience. Using the normalisation process theory provided an opportunity to study mechanisms of ‘real world’ acute care clinician challenges as they integrated the CgDp into acute practice settings and consider if and how these led to desired improvements in end-of-life care. NPT constructs informed the analysis of empirical data collected during the implementation period. NPT was chosen for its resonance with the desired outcomes: behaviour change needs to be normalised to become routine practice. The tensions identified with this ideal and the realities of practice allowed us to generate a model to guide further implementation while drawing attention to barriers and facilitators of success.

The principal findings are that while staff described the CgDp as being intrinsically coherent its use was not in keeping with what they considered to be their primary focus of work, that is, getting patients to recovery. Participants found it challenging to hold the tension created by uncertainty of whether a patient might recover or not in mind, preferring to focus solely on so called ‘active management’ which they defined as disease focused treatment. The findings confirm other studies for example in intensive care [24] and other areas of the UK health service [10], including hospital doctors [25] and Italian general medicine settings [26]. Additionally, some participants had insight into their difficulties in recognising a dying patient (which is not surprising given the challenges all clinicians experience accurately predicting prognosis [27]). Taken together these factors meant the CgDp would be used late, if at all, as it was only of use once agreement was reached that a person was dying. However, if this point was reached the CgDp was praised for offering a more systematic (and in fact, active) approach to care for the dying patient.

There was evidence of the tool being used as a prompt for collaborative working and in some cases junior medical and nursing staff reported being empowered to raise concerns that a patient might be dying with senior medical staff. In turn, however, senior staff would seek advice and reassurance from the palliative care team and sometimes redelegated the completion and hands-on application of the tool back to junior staff. An unintended consequence of the implementation was that closer working between nursing and medical staff left AHPs to feel excluded and their contributions to EOL care unrecognised. This finding demonstrates a need to further foster effective interprofessional collaboration [28].

Perceptions of extensive paperwork and an expansion of roles, including increased activity outwith professionals’ comfort zones, acted as barriers to prospective planning for end of life care and use of the tool was often delayed or incomplete for these reasons. Whilst everyone could contribute, it was possible to opt out from doing so by choosing to focus on other activities and respond to different pressures when prioritising workload. System factors also prevented close working relationships with the palliative care team, and the tool was not integrated into the electronic notes system meaning its use could easily be overlooked. These challenges, the importance of integration of palliative and medical care team working and lack of integration of care of the dying plans with electronic notes, are in keeping with the findings of McConnell et al. [10] and Raijmakers et al. [29], respectively. Thus, for effective implementation and normalisation of EOL care excellence to be achieved, these organisational and systems barriers need to be addressed.

Crucial to the implementation success was the potential for staff to learn from their experiences. Time, opportunities, and lack of skilled facilitation for reflection and case review meant this was not capitalised upon. These experiences need to move beyond provision of education and training, which can be challenging for practitioners to attend [26], towards generating embedded real time practice-based learning experiences [30].

In summary, implementation occurred but with risks such as the lack of recognition of dying leading to unmet needs, and staff experiences leading to unintended learning. As identified in the UK, there is a risk of the tool taking on a life of its own without professionals learning the foundational principles of good palliative care alongside treating potentially reversible conditions.

### Strengths and limitations

This study investigated the real-time perceptions and experiences of interprofessional team members working in an acute medical ward concerning embedding of high quality palliative care. Use of NPT as an analytic framework enabled an understanding of how care of the dying practices were or were not becoming normalized within an acute setting, identification of barriers and enablers to provisions of care excellence and recommendations for learning strategies to support EOL care excellence (see implications section and Table 3). However, the study took place in a single acute care setting within a tertiary hospital which limits the broader applicability of its findings. Potential transferability is enhanced by rich descriptions, using purposive sampling and the findings resonance with existing literature from other settings [20, 31]. The study relied on individual accounts of EOL care practices and engagement with CgDp rather than observations. To increase the study’s dependability [31], data were collected until saturation, no new themes, and data were analysed iteratively by an interprofessional team. Further strategies to enhance study credibility included seeking feedback from participants on our data analysis. There would have been value in also having the middle range programme theory model confirmed by further professional validation. This was not practical; however, the model has been informed by NPT and generated through extensive, reflexive discussions within the research team. Finally, to quantify outcomes, there may have been value in conducting a chart audit to determine when the CgDp had been used, and with what effects, however, our goal in the present study was to primarily explore the process of implementation.

### Implications for practice and further research

This study provides important empirical evidence from healthcare practitioners working in acute care settings on their experiences of providing EOL care. It is an example of patient focussed theoretically informed medical education research that extends into clinical practice [32]. The findings and conceptual model, could inform and optimise interventions to support practitioners’ effective normalisation of EOL care excellence in acute care settings. EOL care excellence requires meaningful collaboration with and specialist support from the palliative care team combined with effective interprofessional co-working [33]. As the acute care team’s coherence, collective action, cognitive participation and reflective monitoring are enhanced, the reliance on specialist palliative care may be limited to complex cases.

To achieve these goals in practice, we propose the following learning strategies informed by the NPT constructs and our findings (See Table 3 and Fig. 1). To address coherence, acute care staff need support to develop their palliative care knowledge and skills to recognise dying and the point at which to initiate the pathway whilst developing the ability to hold two approaches to care at the same time during transition [21]. This must also relate to understanding of the importance of integrating care practices and promoting the CgDp as a positive aspect of care rather than a failing (See Fig. 1). Expert and accessible guidance (See Fig. 1) e.g. from palliative care specialist or champions is required to assist acute care staff to navigate such situations of clinical uncertainty e.g. case-based interprofessional and interdisciplinary team learning sessions.

Learning strategies to increase cognitive participation might include harnessing genuine interprofessional working (See Fig. 1) to promote shared understanding of each other’s roles (see Table 2) [34]. Furthermore, learning strategies might include creating meaningful interprofessional learning experiences where collaboratively the team work through genuine cases to make decisions on EOL care and to identify team members’ roles and responsibilities (See Fig. 1); or learning sessions with palliative care team members who provide guidance on collaborative approaches EOL care (See Table 3).

To address collective action, consideration needs to be given to reviewing work structures, rostering and processes to support the prioritisation of EOL care e.g. prioritising dying patients on ward rounds; ward-based AHPs (See Table 3 and Fig. 1) [22]. In this regard rearranging key documents and pathways within the hospital care processes can help facilitate delivering high quality end of life care. By such so called “textual regulation” [35], the institution can demonstrate that it values end of life care and support workers. Moreover, creation of learning programs challenging assumptions about work practices facilitated by the palliative care team to role model effective interprofessional co-working (See Fig. 1) may further help [21]. A key system factor to support change would be integration of the CgDP into the routine electronic records.

Finally, strategies (also see Table 2) likely to augment acute care staff’s ability to reflexively monitor EOL care and recognise EOL care excellence include: 1) local quality improvement processes to assist staff understanding of EOL care and provide local solutions to specific barriers and gaps; 2) foster feedback processes [21, 36] to increase practitioners’ ability to self-evaluate EOL care performance and 3) create opportunities for staff to debrief as a team e.g. ‘after death’ care reviews with support from the palliative care team (See Fig. 1).

## Conclusions

Our findings emphasise the importance of in-depth examination of implementation processes and offers strategies for normalising EOL care excellence in acute care setting using guidelines. Further exploration into these experiences will provide insights to how these tensions might be reconciled and/or held to ensure holistic EOL in acute care settings. Finally, research to test and refine our programme theory model is required.

## Abbreviations

AHP: Allied health practitioners
CgDp: Clinical Guidelines for Dying Patients
EOL: End of life
LCP: Liverpool Care of the Dying Pathway
NPT: Normalisation Process Theory
UK: United Kingdom
